# Cancer registries in Europe—going forward is the only option

**DOI:** 10.3332/ecancer.2016.641

**Published:** 2016-05-12

**Authors:** Ana-Maria Forsea

**Affiliations:** Dermatology Department, Elias University Hospital, 17 Marasti Bvd, Sector 1 Bucharest 011468, Romania

**Keywords:** cancer registries, cancer control, cancer burden, Europe, disparities

## Abstract

Cancer registries (CR) are the fundamental source of objective cancer data, and thus are indispensable for the evaluation of the cancer burden and for design of effective cancer control plans. Their potential roles spread far beyond epidemiological research, from the exploration of the causes of cancer to health economics, from the evaluation of mass screening programmes to monitoring the quality and outcomes of health services, from addressing the inequalities in access to healthcare, to patients’ quality of life analyses, from treatment safety to the development of biomarkers.

In Europe, cancer registration is challenged by significant disparities in the quality and coverage of CRs, by insufficient harmonisation and comparability of procedures and data, by heterogeneous legislation that limits CR’s abilities for networking, collaboration, and participation in research. These arise against the background of large variations in economical, regulatory, social, and cultural national contexts.

Important steps have been taken at European Union (EU)-level in recent years towards mapping and understanding these challenges, identifying best practices and formulating sensible recommendations, and creating the policy frameworks and the tools for cooperation and information sharing. Yet, as cancer has now become the second cause of death in Europe, one third of the population still lacks quality cancer registration, mostly in the regions with lowest resources and health status. It is therefore imperative that the efforts to support the development of CRs continue, and that the wealth of knowledge and vision acquired in this area is transformed into action.

## Introduction

Cancer is on the rise worldwide, and it is becoming a global major public health problem. In Europe it is the second cause of death and generates extensive medical and social costs [1]. Effectively fighting this complex disease needs an evidence-based approach, which can only be built on the foundation of accurate and complete data provided by CRs. CRs are the fundamental source of objective information regarding the number of new cancer cases (incidence), cancer-caused deaths (mortality), number of individuals living with cancer (prevalence), and cancer survival rates in the populations they cover. They enable the assessment of the current situation and the estimation of the future trends in the cancer burden in different populations and thus constitute an indispensable knowledge base for the design of all levels of effective cancer control plans–from the identification of high risk groups, to defining the most appropriate interventions for prevention, early diagnosis and treatment; from the planning of resources, allocation for various health care interventions, to the evaluation of the quality and outcomes of healthcare services.

Their role is crucial, but sometimes overlooked. Compared to the glaring disparities in cancer survival or access to treatment across the world, the disparities in cancer registration and data availability make their way less often to the first page of newspapers and to public awareness. The complexity of registries function is often not even visible to the health professionals providing and using their data for research or medical care. Their output is variable and disseminated in a fragmented way, so that comparison and overview is difficult. Their role and benefits are not enough understood by the decision-makers of health-policies. Yet 85% of the world population lacks quality cancer registration [2, 3]. One-third of the European population is in the same situation [2, 4], mostly the less affluent countries. Thus patients see their suffering underestimated and countries, especially the ones most in need, are deprived of the objective tools for guiding their efforts and managing wisely their limited resources for the fight against cancer.

This review aims to outline the situation of population-based cancer registration in Europe, highlighting its challenges and opportunities, in order to support the various efforts involved in improving, expanding, and harmonising cancer registration in this continent.

## Cancer registries: structure, roles, and function

Population-based cancer registries (PCR) are organisations destined to systematically collect, store, analyse, and report the data on all cancer cases occurring in a certain population, usually corresponding to a specific geographical area [5, 6].

They are complex organisations, functioning under the authority of an organisation (programme owner) usually public, which is responsible for the registries’ organisation and funding [7–9]. PCRs need human resources and infrastructure to gather data on cancer cases from a wide range of medical and civil databases. They have staff, logistics, and methodology to store, analyse, interpret the cancer data, and to self-control the quality of their own functioning. CRs need a platform to report and disseminate their results on regional/national and international levels and the human and material resources to link, collaborate, and exchange with various organisations for public health and clinical research. They have multiple stakeholders that employ CRs data for policy decisions, scientific research, medical care guidelines, or for patients rights advocacy. CRs’ function is conditioned by the organisation of the health systems in which they operate and by the legal, economic, social, and cultural dimensions of their specific national and regional context.

The type of data that population-based CRs can provide determines the extent of their role and function. Fundamentally CRs should provide information on cancer incidence, mortality, survival, and prevalence rates, time trends, and projections in the populations covered [2, 5, 6, 8, 10, 11]. On a more advanced plan, CRs can give information on the stage at diagnosis, diagnostic and treatment delay, type of treatment, medical equipment use, and compliance with clinical care guidelines [2, 5, 6, 8, 10–12]. In consequence, CRs have a key role in: i) epidemiologic research, for monitoring the trends of cancer incidence, survival, and prevalence rates in geographical areas, social groups, or time periods; ii) investigation of aetiologic factors for cancer, by supporting the analysis of the impact of different social or environmental factors on cancer risk; iii) the planning of cancer control measures, helping to prioritise different actions according to the current and projected cancer burden; iv) the assessment and monitoring of the effectiveness of cancer control measures: primary prevention, screening programmes, treatment patterns, and health care quality; v) assessment of the impact of differences in access to diagnosis and treatment between geographical areas or social groups, in order to create programmes for reducing health inequalities; vi) clinical and translational cancer research.

These manifold roles can only be fulfilled if CRs can produce data of sufficient quality. The quality of a CR depends essentially on the completeness and the validity of data [3, 5, 6]. High quality registries have a constant concern for internal quality checking [3, 5, 6].

The quality of a CR depends both on its methodology and on the conditions of the health system in which it operates. For a PCR to function, its national context needs to fulfil certain criteria: sufficient quality of the general health care system, to ensure that most cancer cases will be identified and correctly diagnosed; the medical data in the catchment area from hospitals, death certificates etc. should be correctly recorded and stored, and made accessible to registries; reliable population statistical data and clear definition of catchment area.

The lack of trained physicians and histopathologic and imaging diagnostic facilities, or obstacles in patients’ access to diagnostic procedures are major impediments to finding and registering cancer cases. The level of legal reinforcement of cancer registration also has a key role, and it varies largely across countries and health systems, i.e. from the reporting of cancer cases being mandatory by law to no regulation at all [3, 5, 13]. CRs need further to comply with privacy and personal data protection legislation, and this may restrict their linking with other health databases such as vital status or screening programmes [13–15].

Within these context prerequisites, registries’ performance depends eventually on their methodologies, including the sources of data, the set of data collected about each cancer case, and the active or passive method of data collection.

The sources of data on cancer cases usually include: treatment and diagnostic facilities (oncology centres or hospital departments, pathology laboratories, or imaging facilities etc.) and the official territorial death registry. Additional data sources like ambulatory and private clinics, elderly care homes and general practitioners networks increase the completeness of data, but also the logistics and expenses. The extent of available resources and the quality of data transfer between medical facilities and CRs dictate the choice of active versus passive ways of data collection [3, 56]. Similarly, the set of data that each CR collects depends on the quality of medical records sources and on the resources that CRs can deploy for collection. A balance needs to be maintained between the number of variables collected and the ability to ensure the completeness and validity of data [3, 6, 8, 16, 17]. The minimum set of data recommended to be collected by all PCRs was formulated by the International Agency for Research on Cancer (IARC) [6] and by the European Network of Cancer Registries (ENCR) (Table 1) [16]. Additional data (Table 2) [16] should be collected when possible, thereby increasing CRs role in broader domains of research [5, 6].

## Cancer registration: European initiatives

CRs have been expanding in Europe since the early 1900’s. Over the last three decades cancer registration has become an important element of the EU’s strategy against cancer, promoted within the framework of the European Action against Cancer Programme (1985–2008), the European Partnership for Action Against Cancer (EPAAC) (2009–2014) [18], and currently under the EU Cancer Control Joint Action (CANCON) [19]. While cancer has become the second cause of death in Europe, the EU endeavours to achieve pan-European standards in controlling cancer and reducing the tumour burden. Towards this aim, several major steps have been taken at the policy and funding levels to promote CRs development (Table 3).

These pan-European undertakings contributed decisively to reinforce the importance of CRs for cancer control in Europe, to set the policy framework for the CRs function, and to formulate the blueprint for the actions needed to optimise and harmonise cancer registration in the EU. At the same time, they highlighted the challenging disparities in CRs situation and performance across Europe, against the background of highly heterogeneous national contexts.

## Cancer registries in Europe: current situation

At present, nearly 200 PCRs are active in Europe and they are members of ENCR [20]. Together they cover about 60% of the European population with an increasing trend [13]. Quality registration coverage of the entire population by national cancer registries is available in 22 European countries [13, 21, 22]. High quality registration of 10–50% of the population is available in France, Italy, Switzerland, Spain, Germany, and Serbia [22]. High quality registration of <10% of the population is available in Poland and Portugal [22]. EU member states Romania, Greece, and Hungary had as per 2012 only regional or partial data [13, 22, 23], although legislation is in place to allow national cancer registration. For nine European countries (Albania, Bosnia-Herzegovina, FYRO Macedonia, Greece, Hungary, Romania, Portugal, Moldavia, Serbia) the latest national incidence data publicly available for 2012 represent only estimates calculated based on the data available from partial registration and neighbouring countries registries [4, 22].

**Data output.** Comparative analyses of CRs output data constantly reveal a gradient in data quality and complexity across Europe, with Nordic CRs regularly reporting the highest performance, and multiple setbacks recorded in Eastern European countries. The ‘Overview of Cancer Registration in Europe’ [13], gathered information from 99 general PCRs in 32 European countries and revealed that 92% of respondent CRs routinely reported cancer incidence rates [11, 13], 79% contributed to mortality data, and 60% reportedly collected follow-up data for estimating cancer survival rates. The lowest percentage of CRs reporting on survival rates was documented in the South-Eastern European (SEE) region [13], the same region that also reports the lowest survival rates in Europe, down to half of the European average for some cancers [23–25]. As cancer survival rates represent an implemented indicator within the European Core Health Indicators System (ECHIS) [26], its importance warrants special attention for improving the CRs abilities to collect relevant data for its calculation.

The CRs contribution to more advanced health indicators was significantly lower all over the continent. The data supporting the development and evaluation of national cancer control strategies was reportedly produced routinely by 18% of responding CRs and by 58% on a project basis [13]. Only 10% of CRs regularly supported the clinical audits of primary treatment, waiting times, and multidisciplinary of care, while 30–40% of CRs supported these audits on a project basis. The highest percentages were reported in the North-West region [13].

The adherence to treatment guidelines and the impact of the guidelines on patient management were evaluated routinely in 8% and *ad hoc* in about 50% of CRs, with the lowest percentage reported in South-Eastern European countries [13, 27].

Evaluation of mass screening for cancer was regularlysupported by 44% of all responding CRs, but by less than 20% of the CRs in SEE. About 40% of all responding CRs contributed to this evaluation on an *ad-hoc* basis [13, 28].

Data on cancer stage were collected in 61% of the responding registries, varying from 60% in South-West region to 100% in North-West region [13]. Data on first treatment were reported in average by 43% of cancer registries [29].

‘Stage at diagnosis’, ‘cancer treatment delay’, and ‘compliance with cancer management guidelines’ were indicated by EUROCHIP studies as essential indicators for the assessment of cancer burden and care [30]. Consequently, a specific analysis on the CRs performance in producing data for the estimation of the three indicators was performed by EUROCHIP in collaboration with ENCR and EUROCOURSE [31]. The survey-based analysis included 86 general PCRs in 32 European countries and revealed that only 15% of all the CRs in EU were able to produce all three indicators. The indicator ‘stage at diagnosis’ was collected for at least one cancer site by 81% of CRs, while less than 40% of them used the TNM classification for this purpose [21]. About one-third of CRs collected the variables for the indicator ‘cancer treatment delay’, while 15% of the CRs were able to contribute to the indicator ‘compliance to guidelines’. Insufficient access to data sources and lack of qualified staff were the major reasons indicated for not collecting all the variables.

**Methodology and data quality.** European PCRs collect their data from a wide range of sources. Virtually all CRs reportedly collect data from pathology reports. A total of 93% of them collect from hospital records and discharge diagnoses, 83% from radiotherapy departments, and 78% of them also use death certificates [11]. Additional sources like haematology laboratories, hospice/palliative care records, public or private hospital outpatient records, general practitioner records, and health insurance records are variably used by some CRs [3, 29].

Both active and passive methods of data collection are used by European CRs. Passive registration is mostly used by registries in North-Western Europe, where cancer case reporting is also mandatory by law and facilitated by the wide use of digital records. Active registration is more frequent in Central and Eastern European countries. In countries with multiple regional CRs, different methods or combinations may be used, as in Italy, France, or Switzerland [3, 13, 32, 33].

The CRs concern for quality control and the methods used for evaluating the parameters of data quality like completeness and validity are highly variable across Europe. In 2011, the EUROCOURSE survey [34] analysed the responses of 116 of the 179 European general cancer registries, covering 280 million inhabitants of 32 European countries. A 12% of all responding CRs reportedly did not conduct any completeness assessment, mostly because of lack of resources. The remainder of CRs perform routinely or at least occasionally completeness evaluation, but continuous monitoring of completeness occurs only in a minority of CRs. The newest, complex, software-based methods of evaluation are used by a minority of CRs [13, 34]. The results of completeness evaluations are made public in peer reviewed journals by 11.6% of CRs, but are not published anywhere by one third of CRs [34]. Completeness and timeliness of publishing the data tend to be inversely correlated. The median duration of completing data for incidence within last year was 18 months, but this again was with large variations. These results prompted the expert consortium of EUROCOURSE to formulate a set of recommendations for improving and harmonising quality control by CRs [34, 35], and the JRC in cooperation with ENCR has very recently released a first version of a software for standardised quality-check for ENCR member CRs [20].

**Programme owners and funding.** Programme owners (PO) of CRs in Europe are as diverse as the national contexts. They are mostly public organisations, including: governments, parliaments, and public authorities (ministries of health or sciences, regional health authorities), national institutes of public health and agencies for cancer, national research councils or agencies, health insurance companies, cancer societies, or comprehensive cancer centres [9]. The role and tasks of POs are vast, as they are ultimately responsible directly or indirectly for the existence, continuity, and quality of CRs, for ensuring their funding, legal compliance, development and function within the continuum of national and European cancer control plans [7–9]. EC-funded projects have developed a multitude of support materials for the information and guidance of POs, such as the ‘10 Commandments for Good Governance’ or the ‘Cancer Registree’ concept and publications series [7, 11, 36].

Budget is obviously a fundamental factor influencing the quality and performance of CRs. Continuity and stability of data collection and reporting are essential for the CRs function, and the funding fragmentation and discontinuities are major hurdles reported by a significant proportion of European CRs. According to a joint EUROCOURSE, EUROCHIP, and ENCR survey [9, 11, 29], at European level 88% of the CRs budget are provided by governments, completed by small contributions (5–10%) from competitive research grants, health insurance companies and cancer societies. However these proportions varied significantly by region, and the SEE registries reported the lowest implication of government funding (64%), along with higher than average contributions from health insurance companies (9.1%), competitive research grants (11%), and societies/charities (13%) [7, 29].

The budget available to CRs per new cancer case averaged 150 EUR in the North-West region (Nordic countries, UK, and Netherlands), 183EUR in Central Europe (Austria, Belgium, France, Germany, Luxembourg, Switzerland), 106 EUR in South-Eastern countries (Bulgaria, Hungary, Romania, Albania, Bosnia-Herzegovina, Croatia, Montenegro, Serbia, Slovenia, Turkey, Cyprus, Greece) and only 34 EUR in Eastern Europe (Belarus, Czech Republic, Poland, Russia, Ukraine, Slovakia, Estonia, Lithuania) [29]. These figures need to be considered in the context of national healthcare budgets, as total healthcare expenditure in Europe varies largely from 358 EUR/inhabitant in Romania to 5500 EUR/inhabitant in Luxembourg as for 2012 [37]. The EUROCOURSE recommendation for all countries to have a wide/full funding coverage plan for quality registration is thus still a way off from materialising.

**Legislation.** Cancer cases reporting is mandatory by law in most of the European countries with high quality registers that included in the cancer incidence in five continents series [3]. Few exceptions exist [13, 33], still cancer reporting remains mandatory *de facto* as it is required by the regulations for healthcare quality evaluation. In several countries with multiple regional CRs, cancer may be reportable in some regions but not in others, like in Italy, Spain, or the UK [3].

Personal data protection legislation has a major impact on CRs functioning. In a pan-European survey within the EUROCOURSE project, 20–35% of responding CRs reported legal-related barriers to cancer registration across most of Europe, while in the South-West region these barriers amounted to up to 60% [13, 33]. Particularly concerning are the nationally variable barriers in the linkage of CRs with other health-related databases like mass screening programmes, biobanks, vital status, and causes of death databases [12–14, 33].

**CRs reporting pathways** are diverse across Europe. The ENCR portal [20] provides an overview of active European CRs and their contact details. Each member CR publishes reports or factsheets, at a regional or national level, with different levels of public access. Some of these reports are in the national language only and the delay in publication may be several years. The peer-reviewed publications of registries data vary widely from 0 to over 100 for some CRs [7, 11, 13, 29]. Centralised data at a European level, with an endeavour for comparability and standardisation, is made available through different platforms (Table 5), following recurrent calls for data by ENCR and other major international organisations like IARC.

Additionally, a large number of high resolution studies collected and compiled the data from European CRs, to investigate specific aspects of the cancer burden, outcomes or risk factors in Europe (Table 6). These network collaborations faced the challenge of disparities in the quality and availability of data across the continent. Nonetheless, they produced essential information on the cancer epidemic in Europe, published in high-impact scientific journals. They highlighted the value of CRs for a wide range of research fields including health economics, patients’ quality of life and reported outcomes, patterns and outcome of care, rare diseases, oncogenesis, prevention, treatment safety or biomarkers.

## Cancer registries in Europe: special challenges

**European disparities.** All mapping efforts of cancer registration in Europe point to significant disparities in terms of CRs population coverage, data quality, and data output across Europe, against the background of significant heterogeneity of healthcare systems configuration, legal landscape, and economic situation on the continent. The most signals of setback come from countries in the eastern half of Europe, classified under South-Eastern Europe [13], or Southern Europe [38], Central and Eastern Europe [22], or Eastern Europe [38]. Countries like some former Yugoslavian republics, or Romania report low population coverage and slow development of CRs, insufficient quality of data, and lowest contribution to the generation of Core Health Indicators [2, 13, 23, 31, 34, 35]. Eastern Europe in its various definitions contains eight European countries with only estimated cancer incidence rates available [22] and has the lowest representation in European high resolution studies [23, 39, 40]. The cancer reporting from this region usually produces a skewed image, with strikingly lower incidence rates, but comparable mortality and dramatically lower survival compared to Western Europe [24, 32, 41–43]. The CRs cannot fulfill their mission of reflecting accurately the cancer burden and of supporting effective cancer control planning in the very countries with the biggest challenges of healthcare financing [37].

The causes for these problems are increasingly investigated and show significant overlaps across the region [7, 32, 44]. The core complaints relate to the lack of funding but also to the lack of CRs visibility and also lack of understanding from public health authorities and policy decision-makers of the importance, roles, and benefits of CRs. CRs often lack political and administrative support, and hence their funding, structure, and governance are fragmented and often arbitrarily changed, and regularly cut back. CRs also appear to lack visibility for the other stakeholders, including health professionals, and patients’ organisations. Logistical obstacles are reported such as the lack of trained and constant staff, the lack of essential IT support, but also the excessive bureaucracy and the instability of the legal framework that hampers data collection and linkage with other health databases [7, 32, 44]. Contrasting with the stability and continuity of development in North and Western Europe, Eastern CRs also had to overcome the disruptions and restructuring of health systems following the political transformations over the last decades.

**Legislation.** The functioning of CRs is subject to the legislation for personal data protection. In the EU this has been regulated by the European Directive on Data Transfer (95/46/EC) whose translation into national legislation and implementation is the resort of each member state [14, 33]. Consequently, large differences exist across Europe in the manner and strictness of the Directive’s implementation related to public health research [33], creating large barriers to cancer registration and cancer control monitoring in some countries [45]. The fragmented and heterogeneous legal context at EU level hinders data sharing, cross-borders collaborative research, and important EU initiatives like the ECHIS. Important changes in this field are expected as the European Commission will replace the Directive by the General Data Protection Regulation [46], which unlike the Directive would be binding by itself for all member states. This would imply a harmonisation of data protection regulation across EU, and important concerns are now rising that this harmonisation, if skewed to the restrictive, excessive regulation side, would actually impede even more cancer registration and public health research [13, 14].

**Information fragmentation.** Efficient pan-European cancer registration and cancer burden analysis is hampered by the fragmentation, dispersion, and redundancy of available information. CRs report data on multiple platforms at regional, national, and international level. While several platforms supported by ENCR or IARC present centralised data from most EU countries with efforts of standardisation (Table 5), some CRs only report through regional or national reports in the national language, with variable delays, and with no peer-reviewed publication. The high-resolution studies using European CRs data publish their results in different scientific journals, with different levels of public access restriction (Table 6). Besides the CRs data, the relevant statistics for cancer control, from population statistics to healthcare systems indicators are reported fragmented across multiple databases like Eurostat and OECD, EUROCOURSE, and ECO, ENCR, EUROCHIP and ECHIS, along with the research projects repositories. The mapping efforts as well as the resulting guidelines and recommendations related to CRs function are reported dispersedly across the websites and data repositories of different EU institutions, programmes, and projects (Table 3).

## European CRs: opportunities and future directions

Cancer registration in Europe has progressed constantly over the last decades and the trend is continuing. Since the creation of ENCR in 1990, and boosted by the European Council Recommendations of 2008 [47], the numbers of PCRs have increased to close to 200 at the present, population coverage increased to close to 60%, and cancer registration has been established virtually in all European countries. The organisation and long-standing performance of registries in North-Western Europe are valuable models [48]. The former communist countries have slower progress as they are still facing multiple challenges. The efforts are likely to continue at the European level to increase the visibility of CRs function, to expand the range of data they collect, and their involvement in an increasing range of fields of clinical and translational research [8, 12, 28]. Considerable effort has been put into mapping and understanding the challenges and disparities of cancer registration across the continent, and policy steps are taken to progress towards a harmonised, collaborative, and effective system of cancer surveillance in Europe. The speed of this progress depends on several future developments.

First, as one sees in the EU the legal basis for action in the field of health falls mainly on the member states [49], this is the level where policies need to be amended, and best practices validated by pan-European studies need to be implemented. The evidence basis and extensive guidelines exist. The political will must now be garnered to take the corresponding action in each member state. For this, stakeholders at a national level, from patient advocacy groups to health professionals, from the research community to the general public ought to understand the need for accurate cancer registration and to push this issue upward on the countries’ policy agenda.

This is particularly relevant to the situation of Eastern-European CRs and several solutions have been proposed at the EU-level [8, 9, 28, 36, 44]. CRs in this region are encouraged to engage with all their stakeholders and to actively seek to increase their visibility both to civil society and the medical profession. Patients, the public, physicians, and researchers need to understand that CRs data are their data too, as any initiative to improve healthcare requires an evidence basis that only CRs can provide. CRs should also actively seek trans-borders collaboration for research, to increase their visibility and impact, and should take advantage of the extensive know-how and software-based tools made available through ENCR, as well as of the EU financing tools.

On a top-down approach, efforts need to continue at an EU-level to emphasise to national policy-makers the necessity and urgency of CRs development, and to provide policy and funding support. The accomplishments of EPAAC [18] and CanCON [19] should be continued through the future EU Health Strategy, in order to maintain without disruption the platform for information sharing, best practices identification and stakeholders’ networking and cooperation. Moreover, a set of measurable standards should be considered, in order to concretely assess progress made, identify points of lagging, and prompt member states to allocate resources and action where they are most needed. Moving on from mapping to benchmarking would be the necessary step forward to sustain real progress in CRs development.

ENCR has joined JRC to promote harmonisation of CRs data quality through the development of software tools and CRs guidelines. The same collaboration has just launched the project: Incidence and mortality in Europe, a new standardised and comparable database for monitoring cancer incidence and mortality in the EU. This project is a step towards the implementation of a European Cancer Information System (ECIS) [20]. The establishment of ECIS is a major undertaking, whose completion in the future will achieve major progress in overcoming the current fragmentation of data relevant to the cancer burden and control across European databases. The unified ECIS will be essential also for the smooth advancement of Europe in the era of e-health and health ‘big data’.

This project’s success will depend on the legal environment and the EC’s course on the implementation of the General Data Protection Regulation, which is being widely debated at present and will impact significantly on this area.

## Conclusions

CRs have a fundamental role to play at all levels of effective cancer control programmes. Their objective data are indispensable for understanding and assessing the current cancer epidemic, and for designing, planning, and evaluating optimal measures to oppose it. Demographics and survivorship patterns change, cancer care advances in the area of molecular diagnosis, targeted therapies and personalised interventions, healthcare systems adjust to accommodate mass screening programmes and comprehensive cancer care, while transitioning to e-health and ‘big data’ health—in this context, the role of CRs will expand even further.

In Europe the progress that has been made deserves to continue, and the current momentum of attention and support for quality cancer registration should not be lost. The reduction of disparities in the quality and function of CRs is a cornerstone, as are the efforts to harmonise, standardise, and bring together in a comparable and understandable way the wealth of cancer data across the continent. Wise policies and regulation, engagement of all stakeholders that need to understand their stake, solid collaboration, and above all the will to put into action all the invaluable knowledge acquired will be needed for success.

## Conflicts of interest

None.

## Funding of publication

None.

## Figures and Tables

**Table 1.** Minimal data set for collection by cancer registries—ENCR recommendations [16].

Essential variables: Item	Comment
**THE PERSON**	
Personal identification	In some countries a unique ID number, in others full name combined with date of birth and sex
Date of birth	Given as day, month and year (dd/mm/yyyy)
Sex	Male (M) or Female (F)
Ethnic group	As the population mixture increases this variable will increase in importance also to study inequality. [May be difficult to agree a classification which can be applied across the whole of Europe]
Address including postal (or zip) code	Needed for ID purpose and for geographical based studies
Vital status & date	It may be of value to indicate whether known or assumed (e.g. based on linkages to death certificates) (dd/mm/yyyy)
Date of death	Needed to study survival and follow-up (dd/mm/yyyy)
Last follow-up date	Needed to study follow-up (dd/mm/yyyy). Registry should indicate whether date refers to active or passive follow-up.
**THE TUMOUR**	
Incidence date	This date should be given priority as outlined by the ENCR recommendations as indicated here A–D. (Optional: In order to have comparability more dates should be collected, preferably all included in the definition)
A: Date of first histological/cytological confirmation of the tumour	Date of biopsy or date of pathology or date of pathology report (dd/mm/yyyy)
B: Date of first hospital admission or contact	May be the date of first out-patient visit for the disease (dd/mm/yyyy)
C: Other date of diagnosis	e.g. GP visit (dd/mm/yyyy)
D: Date of death	For cases discovered at death/autopsy or unknown (dd/mm/yyyy)
Primary tumour site	This should as a minimum be according to the ICD-O (International Classification of Diseases for Oncology)
Laterality	This should be recorded for all paired organs, but as a minimum for breast, eye, ovary, testis, and kidney (but observe the multiple primary rules)
Primary tumour histology	This should as a minimum be according to the ICD-O

**Table 2.** ENCR recommendation for optional set of data for CRs (2005) [16].

Optional variables: THE PERSON	
Occupation	Since most cancer patients will be pensioned it should be the longest/last occupation if not a full occupational history is available
Industry	
Marital status	At the incidence date
Smoking status at diagnosis	Current, ex-smoker, non.smoker
Causes of death	Underlying, plus contributing (can be generated by record linkage)
Place of death	
**THE TUMOUR**	
Mode of detection	Especially if screen detected as part of programme
Therapy details	Type of surgery Chemotherapy regimes Radiation fields – and radiation type Specification of endocrine therapy Record whether the given therapy was intended to be curative or not
Differentiation	As indicated in the ICD-O manual
Grade	For bladder tumours – grade at date of diagnosis
Recurrence	dd/mm/yyyy
Metastasis	Site of metastasis and date of diagnosis (dd/mm/yyyy) or as minimum – local, regional or distant metastasis and date of diagnosis (dd/mm/yyyy)
TNM – full, FIGO, Ann-Arbor etc.	If the registry has easy access to the full TNM or other stage classifications these should be recorded
**FOLLOW-UP**	
Follow-up	Clinical follow-up information–quality of life
Rehabilitation	Active programme/activities should be recorded
Palliation	Palliative activities should be followed

**Table 3.** Main EU initiatives supporting the development of CRs.

Date	Initiative	Role/effects related to CRs
1990	Establishment of the **European Network of Cancer Registries (ENCR)** under the European Commission’s Europe Against Cancer Programme, jointly with the Association of Nordic Cancer Registries (ANCR), the International Association of Cancer Registries (IACR), the Latin Language Registry Group (GRELL) and the International Agency for Research on Cancer (IARC) [11, 20].	• ENCR brings together European cancer registries as members • Promotes quality cancer registration in Europe, the use of cancer data for public health/clinical research, and the continuous monitoring of cancer burden. • Supports the member CRs through evaluations, recommendations, and support and training for CRs staff [10, 19]. • Collects data from cancer registries and reports cancer data through its own platform (EUROCIM) or in collaborations (EUROCOURSE, EUROCARE [11, 23, 50] etc.)
2008	The **European council conclusions for the new European Health Strategy** [47, 51] indicated that EU member states should develop National Cancer Control Programmes, in which the establishment of Cancer Registries was included as statutory requirement.	Stimulus for the establishment of cancer registries in many countries, especially in new EU member states.
2001–2012	**EUROCHIP** (European Cancer Health Indicator Project) projects 1–3 [52], funded by DG Sanco aimed to address the inequalities in cancer by improving information and knowledge on cancer.	Multi-stakeholders network of cancer experts and organisations who gathered and compared across Europe the information necessary to build the base of a future EU common cancer control plan.
	**EUROCHIP 1** (2001–2003) defined the list of cancer health indicators necessary to describe cancer risk, care and survival in Europe to be included in the European Community Health Indicators framework.	Recommended list of indicators related to cancer registries (Table 4) [30]
	**EUROCHIP 2** (2004–2006) identified the gaps in knowledge at countries- and EU- level that impacted on the inequalities in cancer surveillance and control.	**‘Recommendations to the network of competent authorities to support cancer registries’ [53]**
	**EUROCHIP 3** assessed the capacity of European CRs to provide the data necessary for the cancer health indicators;	Mapped the major problems reported by the CRs [31]
1995–2012	Elaboration of the **European Core Health Indicators (ECHI)**, by the European Commission in collaboration with member states, and multiple international organisations	• A 88 indicators list serving as benchmark for effectively monitoring health and health systems in EU [26]. • Indicators derived from CRs are: ‘cancer survival rates’ (implemented) and ‘cancer treatment quality’ (under development) [26]. • EU health strategy foresees further support for MS to produce reliable data for ECHI.
2009	**‘Analysis of National Cancer Control Programmes in Europe’** (2009) by R. Atun *et al* [15], supported by the European Cancer Organization (ECCO)	• The first comprehensive analysis of National Cancer Control Programmes in Europe. • Revealed significant disparities and gaps in cancer registration across Europe.
2009–2012	**EUROCOURSE** (Europe against cancer: Optimisation of the Use of Registries for Scientific Excellence in Research) project [50], initiated by the ENCR and their stakeholder paymasters, funded under the 7th Framework Programme of the Directory General Research of the European Commission.	• Aimed to improve the use of CRs in European countries, through networking, information exchange and benchmarking of best practices. • Mapping of the activity, contexts and challenges of cancer registries in Europe. • Produced comprehensive, knowledge-substantiated recommendations for improving the different aspects of CRs function.
2012	Initiative for a united **European Cancer Information System (ECIS)** [10, 54] by EPAAC Joint Action and European Commission’s Joint Research Centre	• Proposal for the harmonization and sharing of cancer data throughout the EU. • Cancer-registries-derived information would play a key role in the future ECIS.
2009–2014	**The European Partnership for Action Against Cancer,** a multi-stakeholder cooperation, co-financed by EC The **‘European Guide for Quality National Cancer Control Programmes’** [8]	• Emphasised the crucial importance of PCRs as providers of objective and standardised information on cancer risk factors, patterns of care and patients outcomes. • Reiterated the necessity of establishment of National Cancer Registries, in Member States. • Provided recommendations on the logistical, methodological and legal aspects of setting up quality cancer registries.

**Table 4.** Proposed indicators relevant for cancer, for inclusion in the European Core Health Indicators system, EUROCHIP 1 [10, 30].

Population covered by high quality CR
Cancer incidence rates, trends and projections
Cancer survival rates, trends and projections
Cancer prevalence proportions, trends and projections
Cancer mortality rates, trends, projections and person-years life lost because of cancer

Stage at diagnosis: percentage of cases with early diagnosis and with a metastatic test
Delay of cancer treatment: pilot studies
Compliance with best oncology practice

**Table 5.** Resources for European cancer data.

Resource	Description
**Cancer Incidence in five continents** [3]	• Series published every five years by IARC, presents incidence rates based on *recorded* data from high quality registers world-wide. • Considered an international quality standard for cancer registries. • The last volume (X) published in 2014 for the reference period 2003–2007 covers 42% of Europe population [3].
**Globocan** project of IARC [22, 40]	• Publishes online *estimates* of incidence, mortality and prevalence rates of major cancer types in 184 world countries. • Estimates are calculated from data provided by cancer registries available at IARC and in the Internet public domain, including for countries without own quality registry data. • Latest set of projections available up to year 2012.
**European Cancer Observatory (ECO)** [4]	• Web-based tool for accessing European cancer statistics [4], created in 2012 by Euro course project, joint with ENCR. • Consists of three sections: i) EUREG provides *recorded* data on incidence, mortality, prevalence rates and trends from European population-based cancer registries for 35 major cancers in over 100 populations; ii) EUCAN presents national *estimates* of cancer incidence, mortality and prevalence rates for 24 major cancer types in 40 European countries for 2012, including for countries without own quality registry data iii) EUROCIM is an online platform for downloading cancer datasets from the participating registries, on user-registration- and permission request- base [4, 20].
**European Network of Cancer Registries (ENCR)**	• Provides own overview publications on cancer burden in Europe. • ENCR joined with JRC for publishing a new series of factsheets of Europe-wide data on major cancers [20].

**Table 6.** High resolution studies supported by European cancer registries (selection).

Project	Objective
EUROPREVAL [39]	The first European-wide project that estimated the prevalence of the major cancers in 17 European countries, based on calculations from the CRs data up to 1992.
EUROCARE [55] (European Cancer Registry Based Study On Survival And Care Of Cancer Patients) Projects 1–6 (1989–present)	• Provided an updated description of cancer survival rates and trends in Europe, based on the data provided by quality PCRs. • The latest reports of EUROCARE-5 provide information on cancer survival, prevalence and outcomes of care for 31 countries, from 117 CRs, up to year 2007 and with a planned update for up to 2012–2013.
RARECARE [56] (Surveillance of Rare Cancers in Europe)	Estimate the burden of rare cancers in Europe; Improve the quality and comparability of data on rare cancers across European countries. Network of population-based CRs in 22 European countries
HAEMACARE [57] (Cancer Registry Based Project on Haematologic Malignancies)	Support the epidemiological surveillance of haematological malignancies in Europe, by addressing the challenges of haematologic malignancies coding procedures by CRs and the standardization and comparability of CR data on haematologic malignancies.
EUNICE [58] (European Union Network for Information on Cancer) (2005–2007)	Developed new methods for estimating cancer survival Data from ten longstanding population-based registries
ACCIS Automated Childhood Cancers Information System [59]	Developed by IARC as a continuously updated database of all childhood cancer cases registered in population-based cancer registries in Europe Broad networking of an increasing number of participating cancer registries members of ENCR
ECLIS (European Childhood Leukaemia/Lymphoma Incidence Study) [60], EUROCLUS [61] (European Study on clustering of childhood leukaemia). ECCNA (European Network for Cancer Cancer Research in Children and Adolescents).	High resolution studies in childhood cancers supported by ENCR and European cancer registries
EUROCADET [62] (2005–2009)	Estimate the prevalence and quantitative impact on cancer incidence of major lifestyle-related risk factors, and to provide the European policy-makers an estimate of the potential impact on future cancer burden of different preventive interventions directed at the key avoidable cancer determinants.
EPIDERM [63] (European Prevention Initiative for Dermatological Malignancies) (2008–2012)	Gather and disseminate knowledge on the incidence, risk factors, treatments patterns and illness costs for skin cancers in Europe The project used CRs data as knowledge base for developing best practices recommendations for skin cancer prevention and risk reduction strategies.
EPIC [40] (European Prospective Investigation into Cancer and Nutrition)	Investigates in a prospective manner the relationship between diet, nutritional status, lifestyle and environmental factors, and the incidence of cancer and other chronic diseases. Constitutes one of the largest cohort studies in the world, with over half of million participants recruited across ten European countries, followed for almost 15 years, and building one of the largest biobanks in the world for biochemical and genetic investigations on cancer and other chronic diseases.
